# Effect of eight days bed rest on the incretin effect in healthy volunteers

**DOI:** 10.1186/2197-425X-3-S1-A283

**Published:** 2015-10-01

**Authors:** ST Nielsen, NM Harder-Lauridsen, FB Benatti, A-S Wedell-Neergaard, MP Lyngbæk, K Møller, BK Pedersen, R Krogh-Madsen

**Affiliations:** Rigshospitalet, Centre of Inflammation and Metabolism/ Center for Physical Activity Research, Copenhagen, Denmark; University of São Paulo, School of Medicine, São Paulo, Brazil; Rigshospitalet, Neurointensive Care Unit, Copenhagen, Denmark

## Introduction

Admission to the hospital, and particularly to the intensive care unit, leads to a dramatic reduction in physical activity level, which alters the glucose metabolism towards a pre-diabetic state [1]. Critically ill patients often develop spontaneous hyperglycemia, which is related to an increase in morbidity and mortality [2, 3]. The incretin hormones glucagon-like peptide (GLP)-1 and gastric inhibitory polypeptide (GIP) are secreted from the gut in response to a glucose load and stimulate up to 70 % of the insulin secretion from the pancreatic beta cell in healthy persons, which is named the incretin effect [4]. The incretin effect is reduced in critically ill patients [5], but the underlying mechanism has not yet been determined.

## Objectives

Using strict bed rest as a model of physical inactivity seen during hospitalization, we aimed to determine if bed rest induced physical inactivity would reduce the incretin effect in healthy volunteers.

## Methods

Ten healthy male volunteers (age 22.9 ± 3.6 yrs, BMI 24.5 ± 1.8 kg/m^2^) were included in the study. All volunteers underwent eight days of strict bed rest and received an iso-caloric diet with three meals per day based on an estimation of their resting energy expenditure. DXA scans were performed before and after bed rest to evaluate body composition. Before and after bed rest, all volunteers underwent an oral glucose tolerance test (OGTT) and an intravenous glucose infusion (IVGI) on the following day to mimic the blood glucose profile measured during OGTT. The incretin effect was calculated as the relative increment in total serum insulin response to the OGTT compared to the IVGI.

## Results

Bed rest reduced total lean body mass significantly (p < 0.05) with no change in total fat mass. During the OGTT, blood glucose, serum insulin and serum C-peptide were significantly higher after bed rest (p < 0.05) with no change in the incretin effect (p = 0.6). Bed rest did not change plasma GLP-1 during the OGTT, but plasma GIP was significantly higher after bed rest (p < 0.05). The voluteers became insulin resistant (Matsuda index (p < 0.05)), with no change in beta cell function evaluated by C-peptide derived insulinogenic index.

## Conclusions

Blood glucose and serum insulin were higher during the OGTT after bed rest and the Matsuda index was reduced, indicating insulin resistance. The incretin effect was unaffected. Thus, bed rest does not explain the reduced incretin effect in the critically ill.

## Grant Acknowledgment

CIM is supported by Danish National Research Foundation (DNRF55). CFAS is supported by Trygfonden. This study was supported by grants from The Augustinus Foundation and Aase and Ejnar Danielsen's Foundation. CIM is a member of DD2 (grant no. 09-067009 and 09-075724).
